# Occurrence of Bensulfuron-Methyl Resistance and Target-Site Resistance Mechanisms in *Ammannia auriculata* Biotypes from Paddy Fields

**DOI:** 10.3390/plants11151926

**Published:** 2022-07-25

**Authors:** Longwei Liu, Peng Wan, Yang Li, Zhiwen Duan, Cheng Peng, Shuzhong Yuan, Wei Deng

**Affiliations:** College of Horticulture and Plant Protection, Yangzhou University, Yangzhou 225109, China; llw3052270362@163.com (L.L.); wp2259613278@163.com (P.W.); ly19991020@163.com (Y.L.); dzw709275107@163.com (Z.D.); cpeng@dhu.edu.cn (C.P.); dengwei1990dw@163.com (S.Y.)

**Keywords:** *Ammannia auriculata*, target-site resistance, bensulfuron-methyl, amino acid substitutions, CAPS

## Abstract

*Ammanniaauriculata* is a troublesome broadleaf weed, widely distributed in the paddy fields of southern China. In this study, 10 biotypes of *A. auriculata* were sampled from Yangzhou City, China, where the paddy fields were seriously infested with *A. auriculata*, and their resistance levels to acetolactate synthase (ALS) inhibitor bensulfuron-methyl were determined. The whole-plant response assays showed that nine *A. auriculata* biotypes were highly resistant (from 16.4- to 183.1-fold) to bensulfuron-methyl in comparison with a susceptible YZ-S biotype, and only one YZ-6 biotype was susceptible. *ALS* gene sequencing revealed that three *ALS* gene copies existed in *A. auriculata*, and four different amino acid substitutions (Pro197-Leu, -Ala, -Ser, and -His) at site 197 in the *AaALS1* or *2* genes were found in eight resistant biotypes. In addition, no amino acid mutations in three *ALS* genes were found in the YZ-3 biotype. These results suggested that target-site mutations or non-target-site resistance mechanisms were involved in tested resistant *A. auriculata* biotypes. Finally, a cleaved amplified polymorphic sequence (CAPS) marker was identified to rapidly detect the Pro197 mutations in *A. auriculata*.

## 1. Introduction

Modern herbicides are the primary ways of controlling weeds due to their high-efficiency and labor-saving characteristics, and they have made great contributions to food safety production in the world. However, strong selection pressures have made weeds obtain a powerful weapon in their battle with herbicides—herbicide resistance. To date, there are 267 weed species from 96 crops in 71 countries, which are evolving resistance to herbicides across most sites of action [1]. Target-site resistance due to amino acid substitution in the herbicide target-site genes is one of the most common mechanisms facilitating resistance evolution [2,3,4].

Sulfonylurea herbicides, targeting the acetolactate synthase (ALS) enzyme, are the most important group of ALS inhibitors, and more than 50 active ingredients have been commercialized [5]. ALS inhibitors lead to the death of plants by inhibiting the synthesis of branched-chain amino acids [6,7]. Those herbicides have been widely used in a variety of crop fields worldwide due to their super high effectivity and low toxicity to mammalian species [8]. However, ALS inhibitors have a high resistance risk, and the number of weeds that have developed resistance to ALS inhibitors is the highest among all the different types of herbicides [1]. Resistance to ALS inhibitors can be endowed by several different mechanisms. In most cases, target-site mutations in ALS genes are known to cause the high resistance to herbicides in many field-evolved resistant-weed populations. Nine conserved amino acid substitutions (Ala122 and 205, Pro197, Phe206, Asp376, Arg377, Trp574, Ser653, and Gly654) in *ALS* genes have so far been identified to be closely related to ALS inhibitor resistance [9,10,11]. In addition to ALS mutations, metabolic resistance is also an important mechanism of resistance. Metabolic resistance can evolve independently in resistant weeds, and has been reported in *Sinapis arvensis* L. [12], *Bromus rigidus* Roth [13], *Amaranthus tuberculatus* [14], *Sagittaria trifolia* L. [15], *Ipomoea purpurea* (L.) Roth. [16], and *Bromus japonicus* Thunb. [17]. More often, metabolic resistance and ALS mutations co-occur in the same biotypes, such as in *Papaver rhoeas* L. [18,19], *Amaranthus palmeri* [20], and *Descurainia sophia* L. [21]. Furthermore, a few studies have found that *ALS* gene overexpression can play an important role in resistance to ALS inhibitors in *Cyperus compressus* L. [22] and *Bromus sterilis* L. [23].

*Ammanniaauriculata* is one of the most serious weeds in the rice fields of southern China. In recent years, this species has spread rapidly in Jiangsu, Anhui, and Zhejiang provinces, and poses a serious threat to rice production. Control of *A.* *auriculata* in China relies heavily on the ALS inhibitor bensulfuron-methyl. Many species of weeds in rice fields, including *A.* *auriculata*, have developed resistance to this herbicide [24,25,26,27]. In California, *A.* *auriculata* first evolved resistance to bensulfuron-methyl in 1997 [1]. In addition, Wang et al. [28] reported that 85 out of 88 *A.* *auriculata* biotypes from Zhejiang Province tested by the agar method were resistant to bensulfuron-methyl. However, there is no report about the target-site resistance mechanisms for ALS inhibitors in *A.* *auriculata*. The aims of this study were to: (1) determine the resistance levels to bensulfuron-methyl in 11 *A.* *auriculata* biotypes from Yangzhou City in Jiangsu Province; (2) identify the ALS mutations in underlying resistant biotypes; and (3) develop a cleaved amplified polymorphic sequence (CAPS) marker to rapidly detect resistant *A.* *auriculata* plants with the ALS mutations.

## 2. Results

### 2.1. Bensulfuron-Methyl Dose Response

**Figure 1:**
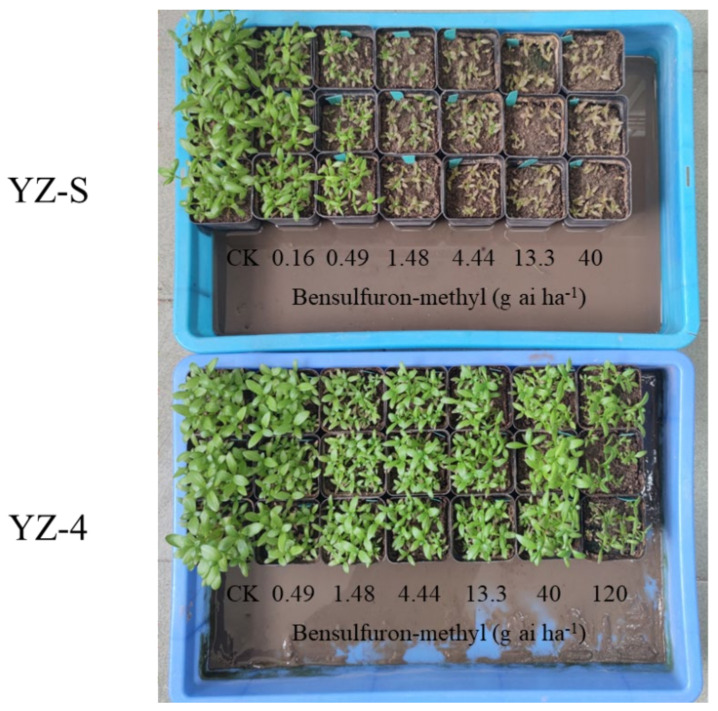
Growth status of YZ-S and -4 A. auriculata plants 21 days after bensulfuron-methyl treatment.

The whole-plant response bioassays indicated that nine A. auriculata biotypes showed the high resistance to bensulfuron-methyl based on the classification of resistance grades (RF ≥ 10) [29]. The GR_50_ values of two susceptible biotypes, YZ-S and -6, were 0.18 and 0.26 g ai ha^−1^, respectively. By contrast, the GR_50_ values of the nine resistant biotypes (YZ-1 to 5 and YZ-7 to 10) to bensulfuron-methyl were between 2.95 and 32.96 g ai ha^−1^, and were from 16.4- to 183.1-fold greater than that of the susceptible YZ-S biotype (Table 1, Figure 1). These results revealed that the bensulfuron-methyl-resistant A. auriculata biotypes were prevalent in the paddy fields.

### 2.2. Identification of ALS Gene Mutations

**Figure 2:**
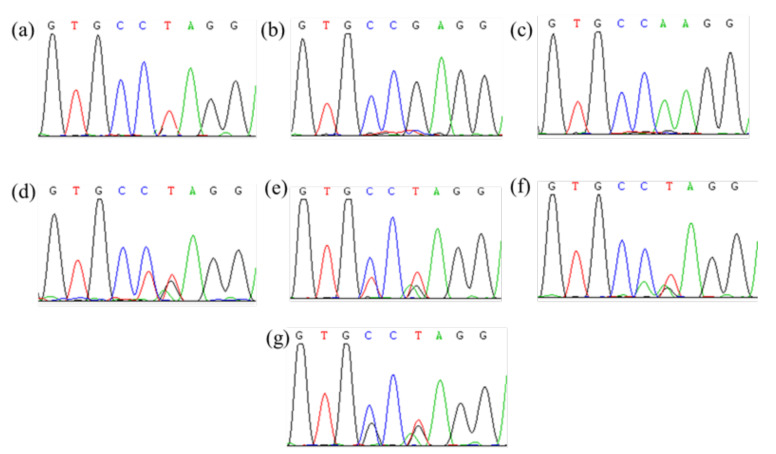
Partial peak diagram of *ALS* sequencing containing Pro197 site. (**a**–**c**) Isolated three *ALS* genes; (**d**–**g**) four different amino acid mutations at Pro197.

A direct sequencing of PCR products showed multiple peaks in certain bases of the chromatogram, indicating the existence of multiple *ALS* gene copies. Three different sequences (*AaALS1, 2*, and *3*) were separated by cloning sequencing. The *ALS* gene sequences of nine resistant *A.* *auriculata* biotypes were compared with those of the susceptible biotypes. Four different ALS mutations (Pro197-Ser, -Leu, -Ala, and -His) in ALS genes were identified in eight resistant biotypes, and no ALS mutations known to endow resistance to ALS inhibitors were observed in the resistant YZ-3 biotype (Table 2, Figure 2). Interestingly, the frequency of resistance-endowing ALS mutations in *AaALS1* was highest in nine tested biotypes, followed by *2*, and no mutations in *3* were observed. These findings indicated that the target-site resistance was the main resistance mechanisms to bensulfuron-methyl, and the Pro197 mutations were very common in *A.* *auriculata*.

### 2.3. CAPS Method for the Pro197 Mutations

**Figure 3:**
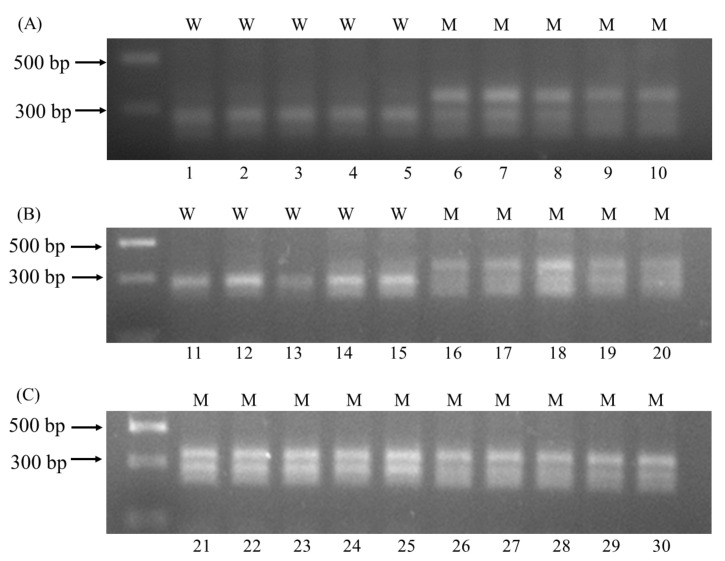
CAPS analysis of 30 *A.* *auriculata* plants carrying wild-type *ALS* alleles (W) or mutant *ALS* alleles (M). (**A**) Lane 1–5 were samples of YZ-S, lane 6–10 were samples of YZ-2; (**B**) lane 11–15 were samples of YZ-3, lane 16–20 were samples of YZ-4; (**C**) lane 21–25 were samples of YZ-8, lane 26–30 were samples of YZ-9.

The *BanI* restriction sequences were: 5′G↓GYRCC3′, and the two cytosine bases were the first two bases of codon 197. Thus, the *A.* *auriculata* plants with or without the Pro197 mutation can be distinguished by product bands after enzyme digestion. The wild-type *ALS* alleles were cut into two fragments of 125 and 200 bp, and the mutant alleles showed three bands of 125, 200, and 325 bp. The YZ-S and -3 biotypes with the wild-type *ALS* genes, and the YZ-2, -4, -8, and -9 biotypes with the mutant *ALS* genes, were selected for CAPS analysis. As expected, the products from YZ-S and -3 plants showed two bands, and products from plants with different Pro197 mutations showed three bands (Figure 3). The results of the CAPS analysis were consistent with the *ALS* gene sequencing results, indicating that the CAPS method can be used to accurately detect the Pro197 mutations in *A.* *auriculata*.

## 3. Discussion

The bensulfuron-methyl resistance levels of 10 *A. auriculata* biotypes from rice fields in Yangzhou City were investigated, and the results showed that 90% of the biotypes evolved resistance to this herbicide. This indicated that bensulfuron-methyl-resistant *A. auriculata* biotypes are widespread in rice-growing areas in China. Indeed, Wang et al. [28] reported that 96.6% of *A. auriculata* biotypes from the Ningshao plain in China were resistant to bensulfuron-methyl. Zhang et al. [30] found that four *A. auriculata* biotypes collected from Zhejiang, Jiangsu, and Anhui provinces exhibited medium to high resistance to bensulfuron-methyl. Bensulfuron-methyl has been used as a pre-emergence or post-emergence herbicide to control broadleaf weeds, including *A. auriculata*, for about 30 years in rice fields in China. The continuous herbicide application may be the driving force of rapid and widespread developments of resistance to bensulfuron-methyl in *A. auriculata*. Thus, herbicides with different sites of action, such as fluroxypyr or bentazon, are recommended as alternative options to control the resistant *A. auriculata*.

In this study, three *ALS* gene copies were identified in *A. auriculata*. Multiple *ALS* genes have been reported in many weed species. For example, *Monochoria korsakowii* and *Monochoria vaginalis* have three and five *ALS* gene copies, respectively [31]. *Decurainia sophia* possesses four *ALS* gene copies [32]. It is easy to miss the target-site mutations in a gene for weed species with multiple *ALS* genes. Thus, it is necessary to detect the sequences of all copies. 

It is widely accepted that target-site mutations are key factors in most cases of ALS resistance [1]. In the present study, four different types of Pro197 mutations were determined in eight out of nine resistant *A. auriculata* biotypes. The results of ALS mutation identifications are strongly associated with those of resistance tests, which demonstrated that the Pro197 mutations in *ALS* play a major role in bensulfuron-methyl resistance in *A. auriculata*. In addition, the Pro197 mutations occurred in either *AaALS1* or *2*, but not in *3*. Similar results were found in other resistant weeds. Xu et al. [32] reported that target-site mutations had a preference for *ALS1* or *2* in four *ALS* gene copies in *D. sophia*. Tanigali et al. [33] found that the resistant mutations exclusively occurred in *ALS1* or *3* in *M. vaginalis*. In our previous study, amino acid mutations in *ALS1* or *2* independently, or in both, were found in *Ammannia multiflora* [34]. The preference of resistant mutations may be related to gene expression levels, herbicide use, and weed species. Interestingly, we found that a *A. auriculata* biotype (YZ-3) showed significant resistance to bensulfuron-methyl, but no resistance-conferring mutations were detected in *ALS* genes, suggesting that other resistance mechanisms may be involved in this case. A further study will investigate the potential resistance mechanisms, such as metabolic resistance.

Several molecular techniques, including PCR-RFLP, CAPS, dCAPS, and LAMP, have been developed for the diagnosis of target-site gene mutations [35,36,37,38,39,40]. Compared with the time-consuming whole-plant response bioassays, these molecular detection methods are rapid, cheap, and reliable, and thus suitable for resistance detections of hundreds of samples. In our study, a CAPS method was established to screen the Pro197 mutations in *ALS* genes in *A. auriculata*. The Pro197-Ser, -Leu, -His, and -Ala mutations in *AaALS1* or *2* can be detected without DNA sequencing. The downside was that plants with non-target-site resistance cannot be detected by this method. 

## 4. Materials and Methods

### 4.1. Plant Materials

We collected seed material of *A. auriculata* from rice fields in Yangzhou City in October 2019. Each sampling location was more than 5 kilometers apart. We collected the known susceptible *A. auriculata* biotype (YZ-S) from experimental fields with no history of herbicide use. We harvested the 10 suspected resistant *A. auriculata* biotypes from rice fields in which bensulfuron-methyl was used for over 15 years (Table 3).

### 4.2. Whole-Plant Response to Bensulfuron-Methyl

We carried whole-plant response assays out to determine the GR_50_ reaction (herbicide dose causing 50% plant-growth inhibition) of 11 *A. auriculata* biotypes with bensulfuron-methyl. We cultivated *A. auriculata* plants in a glasshouse during the growing seasons (May–July; 35/25℃). We used seedlings at four-leaf stage for dose response assays. We applied bensulfuron-methyl (10% wettable powder; Jiangsu Dongbao agrochemical Co., Ltd., Yangzhou, China) at various rates of 0, 0.055, 0.16, 0.49, 1.48, 4.44, 13.3, 40, 120, and 360 g ai ha^−1^ using a cabinet sprayer with a fan nozzle delivering 450 L ha^−1^ at 0.2 MPa. We returned plants to the glasshouse after herbicide treatment. We measured the fresh weight of above-ground part 21 d after treatment. We conducted each treatment in a random design with three replications, and each replication contained at least 12 *A*. *auriculata* plants. We repeated the experiment twice.

### 4.3. ALS Gene Sequencing

We isolated the total RNA from the leaf samples of *A. auriculata* biotypes using the RNAprep Plant Kit (Tiangen, Beijing, China), and we reverse transcribed into cDNA using the Fast RT Kit (Tiangen, Beijing, China). We designed two primer pairs (F1: 5′-GGACATCCTCGTGGAGGC-3′/R1: 5′-TTGTTCTTTCCAATTTCAGC-3′ and F2: 5′-TGTTCAGGAGTTAGCCACGATCAGG-3′/R2: 5′-TTAGTAAGATGTCCGCCCGTCACCC-3′) to amplify the fragment of *ALS* genes. We conducted the PCR reaction in a volume of 50 µL that contained 25 µL of 2×Taq Plus PCR Mix, 1 µL of each primer, 2 µL of cDNA, and 21 µL of ddH_2_O. We ran the PCR using the following cycles: 94℃ for 5 min, 35 cycles of 94 ℃ for 30 s, 58 ℃ (F1/R1) or 62 ℃ (F2/R2) for 30 s, and 72 ℃ for 40 to 60 s, followed by 72 ℃ elongation for 10 min. We purified and sequenced the PCR products from both ends with above amplification primers by commercial services (Sangon Biotech, China). Because there were three *ALS* gene copies in *A. auriculata*, for samples with ALS mutations detected by directly sequencing, we cloned the amplified fragments into the pGM-T vector to distinguish which *ALS* genes were mutated. We randomly selected a total of 10 plants from each *A. auriculata* biotype for identification of ALS mutations.

### 4.4. CAPS Detection for Pro197 Mutations

We used a total of 30 plants from 6 *A. auriculata* biotypes (YZ-S, -2, -3, -4, -8, and -9) for CAPS genotyping. We designed primers (F: 5′-GGTCAGCGGCCTCGCC-3′/R: 5′-TGGCTTGGGCAACCTGGA-3′) to amplify the ALS gene fragments (325 bp) containing the Pro197 site. We performed the procedures in the same PCR conditions described above. Then, we used the amplified products for enzyme digestion. We carried the digestion reaction out in a 50 µL volume containing 1 µL of restriction enzyme *BanI* (NEB, USA), 5 µL of 10×NEBuffer, 10 µL of PCR product, and 34 µL of ddH2O. We ran the reactions at 37 ℃ for 60 min, and inactivated them at 65 ℃ for 10 min. After that, we visualized the digestion products by electrophoresis on 2% agarose gels at 110 V for 50 min.

### 4.5. Statistical Analysis

In the whole-plant response experiments, we converted the data of fresh weight into a percentage of the nontreated control. We evaluated the GR_50_ values using the equation: y = C + (D - C) / [1 + (x / GR_50_) ^b^] [41]. The resistance factor (RF) was expressed by the GR_50_ of the resistant *A. auriculata* biotypes divided by the GR_50_ of the susceptible biotypes.

## 5. Conclusions

In conclusion, 9 out of 10 *A. auriculata* biotypes showed a high level of resistance to bensulfuron-methyl. Herbicides with different sites of action should be used to control the resistant *A. auriculata*. *A. auriculata* plants possess three *ALS* gene copies, and resistance-conferring Pro197 mutations were found in *AaALS1* and *2*. Moreover, we established the CAPS method to detect the Pro197-Ser, -Leu, -Ala, and -His mutations in *A. auriculata*.

## Figures and Tables

**Table 1:** Parameter values of log-logistic equation for whole-plant response assays of 11 *A.* *auriculata* populations to bensulfuron-methyl.

Population	Susceptibility	Regression parameter				GR_50_ (g ai ha^−1^)	RF
		C	D	b	R2		
YZ-S	S	14.68	80.36	1.54	0.99	0.18 (0.015)	1.0
YZ-1	R	11.73	98.61	0.91	0.98	3.18 (0.59)	17.7
YZ-2	R	10.42	105.51	0.74	0.98	3.36 (0.37)	18.7
YZ-3	R	20.79	95.27	2.24	0.98	2.95 (0.60)	16.4
YZ-4	R	14.55	91.15	1.00	0.97	20.97 (3.80)	116.5
YZ-5	R	16.52	85.45	1.20	0.99	4.82 (1.12)	26.8
YZ-6	S	13.05	81.35	1.55	0.99	0.26 (0.02)	1.4
YZ-7	R	19.50	95.01	1.60	0.99	32.96 (4.87)	183.1
YZ-8	R	17.19	84.71	1.76	0.99	7.18 (0.38)	39.9
YZ-9	R	11.78	96.69	1.01	0.99	6.91 (1.68)	38.4
YZ-10	R	15.64	89.80	1.35	0.98	6.50 (1.07)	36.1

**Table 2:** Codon and encoded amino acids at site of Pro197 in *ALS* genes in 11 *A.* *auriculata* biotypes.

Biotype	Susceptibility	Pro197 in AaALS1		Pro197 in AaALS2		Pro197 in AaALS3		Numbers of Plants with Specific ALS Genotype/Total Tested Plants
		Codon	Amino acid	Codon	Amino acid	Codon	Amino acid	
YZ-S	S	CCT	Pro	CCG	Pro	CCA	Pro	10/10
YZ-1	R	**CTT**	Leu	CCG	Pro	CCA	Pro	10/10
YZ-2	R	**GCT**	Ala	CCG	Pro	CCA	Pro	5/10
		**TCT**	Ser	CCG	Pro	CCA	Pro	5/10
YZ-3	R	CCT	Pro	CCG	Pro	CCA	Pro	10/10
YZ-4	R	**CAT**	His	CCG	Pro	CCA	Pro	10/10
YZ-5	R	**CTT**	Leu	CCG	Pro	CCA	Pro	10/10
YZ-6	S	CCT	Pro	CCG	Pro	CCA	Pro	10/10
YZ-7	R	**CTT**	Leu	CCG	Pro	CCA	Pro	9/10
		**GCT**	Ala	CCG	Pro	CCA	Pro	1/10
YZ-8	R	**CTT**	Leu	CCG	Pro	CCA	Pro	10/10
YZ-9	R	CCT	Pro	**TCG**	Ser	CCA	Pro	10/10
YZ-10	R	**TCT**	Ser	CCG	Pro	CCA	Pro	10/10

**Table 3:** Geographical origin of 11 *A. auriculata* populations collected from paddy fields.

Population	Location	Co-Ordinate
YZ-S	Sunongwu Village, Hanjiang District, Yangzhou City	119.4290 E, 32.3983 N
YZ-1	Qingyu Village, Lidian Town, Yangzhou City	119.6708 E, 32.3112 N
YZ-2	Yanjiang Village, Lidian Town, Yangzhou City	119.5778 E, 32.2670 N
YZ-3	Chenhua Village, Fangxiang Town, Yangzhou City	119.4095 E, 32.4990 N
YZ-4	Shiqiao Village, Gongdao Town, Yangzhou City	119.2837 E, 32.5521 N
YZ-5	Wangzhuang Village, Gongdao Town, Yangzhou City	119.4102 E, 32.6226 N
YZ-6	Pantang Village, Guoji Town, Yangzhou City	119.3765 E, 32.6884 N
YZ-7	Qunan Village, Longben Town, Yangzhou City	119.5072 E, 32.7517 N
YZ-8	Xiajiazhuang Village, Xiejia Town, Yangzhou City	119.6263 E, 32.7601 N
YZ-9	Lizhuang Village, Guanglin District, Yangzhou City	119.4113 E, 32.3251 N
YZ-10	Shenzhuang Village, Guanglin District, Yangzhou City	119.3786 E, 32.2763 N

## Data Availability

The data are available on request from the corresponding author.
